# The Antioxidant Effects of Whey Protein Peptide on Learning and Memory Improvement in Aging Mice Models

**DOI:** 10.3390/nu13062100

**Published:** 2021-06-19

**Authors:** Xiao-Chen Yu, Zhen Li, Xin-Ran Liu, Jia-Ni Hu, Rui Liu, Na Zhu, Yong Li

**Affiliations:** Department of Nutrition and Food Hygiene, School of Public Health, Peking University, Beijing 100191, China; xiaochenyu46@126.com (X.-C.Y.); lizhenbjmu@163.com (Z.L.); liuhappy07@163.com (X.-R.L.); hujiani95@163.com (J.-N.H.); lrui_pku@163.com (R.L.); summer920503@163.com (N.Z.)

**Keywords:** whey protein peptide, D-galactose, antioxidation, learning and memory

## Abstract

This study investigated the antioxidant effects of whey protein peptide on learning and memory in aging C57BL/6N mice. A total of 72 SPF male C57BL/6N mice were used. Twelve mice were randomly selected as the control group, and the other mice were intraperitoneally injected with D-galactose (100 mg/kg body weight for 6 weeks), during which, the mice in the control group were intraperitoneally injected with the same amount of normal saline. After 6 weeks, the blood was taken from the epicanthus and the serum MDA level was measured, according to which, the mice were randomly divided into the model control group, the whey protein group (1.5 g/kg body weight), and three Whey protein peptide (WHP) intervention groups (0.3 g/kg body weight, 1.5 g/kg body weight, 3.0 g/kg body weight). The water solution of the test sample was administered by oral gavage every day. The intervention period was 30 days, during which, the model control group, the whey protein group, and the whey protein peptide group continued receiving intraperitoneal injections of D-galactose, while the control group continued receiving intraperitoneal injections of normal saline. After the intervention, behavioral experiments were conducted in the following order: open field test, water maze test, and new object recognition test. After the behavioral experiment, the morphology of hippocampal formation was observed by HE staining and TUNEL labeling. Oxidative stress-related indexes in the serum, liver, and brain were detected. Expression levels of the cholinergic system-related enzymes and proinflammatory cytokines in brain tissue were detected. Western blot was used to detect the expression of synaptic plasticity-related proteins in the mouse brain. The results showed that WHP could significantly improve the accumulation of MDA and PC, increase the activities of SOD and GSH-Px, resist oxidative stress injury, and enhance the potential of endogenous antioxidant defense mechanisms. WHP can significantly improve the decline of aging-related spatial exploration, body movement, and spatial and non-spatial learning/memory ability. Its specific mechanism may be related to reducing the degeneration of hippocampal nerve cells, reducing the apoptosis of nerve cells, improving the activity of AChE, reducing the expression of inflammatory factors (TNF-α and IL-1β) in brain tissue, reducing oxidative stress injury, and improving the expression of p-CaMKⅡ and BDNF synaptic plasticity protein. These results indicate that WHP can improve aging-related oxidative stress, as well as learning and memory impairment.

## 1. Introduction

“Aging or senescence” refers to the gradual degenerative changes in the body (that occur as one ages), manifested as abnormal levels of functional metabolism, a decrease in the ability to adapt to the external environment, and a decline in the ability to resist stimulation damage [1]. According to WHO statistics, the proportion of the global population aged 60 and older is expected to increase from 900 million in 2015 to 1.4 billion in 2030, 2.1 billion in 2050, and exceed 3.2 billion in 2100. The global proportion of elderly people is continuously increasing; thus, the world should pay attention to the various aging-related issues. At present, the mechanism of aging remains controversial. In the 1950s, Gerschman proposed the free radical theory [2], which stated that the damage caused by free radicals to cell components or intercellular components was the main reason for the damage of physiological and biochemical functions related to aging of the body. Under normal circumstances, the cell undergoes a reversible redox state change during the metabolism process and constantly produces free radicals. The body contains endogenous antioxidants, including enzymes and non-enzymatic pathways. The main antioxidant enzymes include superoxide dismutase, glutathione peroxidase, etc. They participate in the scavenging function of free radicals under normal conditions. When the production and accumulation of various endogenous and/or exogenous reactive oxygen species are excessive, or when the antioxidant capacity decreases, the balance between the endogenous defense system and free radicals will be broken, resulting in oxidative stress, which will gradually cause continuous and cumulative oxidative damage to cells. This damage is mostly manifested in potential harm to biological macromolecules, such as lipids, proteins, and DNA. In the body, it is manifested in the accumulation of lipid peroxides (such as malondialdehyde) and carbonylated proteins in tissues and organs [3].

As individuals age, there is, objectively, “brain aging” in elderly people. Brain aging can cause degenerative changes in the nervous system. In severe cases, it can cause mild cognitive impairment, Alzheimer’s disease, or other common diseases associated with the elderly, which are manifested by different degrees of decline in learning, memory ability, and cognitive impairment. Learning and memory ability refers to the process by which humans and animals change their behaviors and activities to adapt to environmental changes based on the experiences they have acquired. The maintenance of cognitive function is (believed to be) related to the interaction and connection between neurons and synapses in the hippocampus and brain. With aging, the oxygen demand in brain tissue continues to increase. As the brain tissue is sensitive to the stimulation of oxidative stress, excessive accumulation of free radicals can easily occur in the brain, which cannot be effectively self-cleared; thus, leading to the damage of neural stem cells and neurons, causing serious damage to normal nerve function, and ultimately leading to the decline of learning and memory ability. Therefore, exploring natural antioxidants to (more safely and effectively) delay brain aging, by interfering with oxidative stress damage, and slow aging-related hippocampal neuron damage (i.e., to improve learning and memory disorders) is essential, in order to achieve healthy aging, and improve the well-being of elderly individuals.

Whey is the main by-product in the production of cheese and casein. It accounts for 85% to 95% of the total milk volume and contains about half of the milk nutrition. It was reported that the annual output of whey exceeds 160 million tons [4]; improper treatment methods will not only cause pollution problems, but also cause the loss of resources. Therefore, the rational processing and recycling of whey has become a popular area of research. The protein component of whey is a high-quality protein. Whey protein contains eight essential amino acids required by humans. Benefiting from various active ingredients, such as β-lactoglobulin, α-lactalbumin and lactoferrin, whey protein has been used in various industries, such as fitness and medical care [5]. Whey protein peptide (WHP) is an enzymatic hydrolysis product of whey protein. As a kind of biologically active peptide, it has the advantages of a high absorption rate, a high utilization rate, it does not typically cause adverse events, it involves low energy consumption, no competitive inhibition, etc. WHP was also shown to regulate blood lipids, and promote muscle protein synthesis and other biological activity. However, there are few studies on the antioxidant effects of WHP in regards to learning and memory in aging individuals. Therefore, the present study was performed to explore the antioxidant effects of WHP, in regards to improving learning and memory functions in aging mice models.

## 2. Materials and Methods

### 2.1. Preparation of WHP Sample

WHP was extracted from the proteins of whey via enzymatic hydrolysis and provided by Tianjin Milkyway Import and Export Co., Ltd. (Tianjin, China). Briefly, WHP is a small molecule bioactive peptide mixture, which is extracted from whey protein concentrate by a special cross flow filtration process, and was then separated from whey protein by enzymatic hydrolysis technology. WHP8350 produced by Hilmar was used in this study. The protein peptide content of WHP8350 was 67.2%, which contains more glutamic acid, aspartic acid, leucine, and lysine. The amino acid composition of whey protein peptide is shown in Table 1.

### 2.2. Chemicals and Reagents

Whey protein and WHP were obtained from Tianjin Milkyway Import and Export Co., Ltd. (Tianjin, China). D-galactose was purchased from Beijing FM Bioscience Co. (Beijing, China), Ltd. Assay kits used for the determination of superoxide dismutase (SOD), glutathione peroxidase (GSH-Px), malondialdehyde (MDA), protein carbonyl (PC), acetylcholinesterase (AChE), and choline acetyl transferase (ChAT) were purchased from Nanjing Jiancheng Biological Engineering Research Institute (Nanjing, China). Tumor necrosis factor α (TNF-α) assay kits were purchased from MultiSciences (Lianke) Biotech Co., Ltd. (Hangzhou, China). Interleukin (IL)-1β assay kits was purchased from Beijing Zhongshang Boao Bio Technology Co., Ltd. (Beijing, China). Phospho-CaMKⅡ (Thr286) antibody and CREB (48H2) rabbit mAb were purchased from CST (USA). Anti-CREB (phospho S133) antibody, anti-BDNF antibody, anti-CaMKⅡ antibody, goat anti-rabbit IgG, and anti-beta actin antibody were purchased from Abcam (Cambridge, UK). TUNEL assay kits and HE dye liquor were purchased from Servicebio (Wuhan, China).

### 2.3. Animals and Experimental Design

C57BL/6N mice (male, weighing 40 ± 10 g, 6 months) in a specific pathogen-free condition were obtained from the Department of Laboratory Animal Science at Peking University (Laboratory animal production license No.: SCXK (Jing) 2016-0010; laboratory animal use license no.: SYXK (Jing) 2016-0041). The mice were kept in a mice laboratory in the Department of Laboratory Animal Science, which was in a filter-protected and air-conditioned room, with constant temperature (21–25 °C), a humidity of 50–60%, and photoperiod of 12 h. Two mice were housed in a cage and had free access to standard food (American Institute of Nutrition Rodent Diets-93G (AIN-93G diet) and water. All experimental procedures were approved by the Peking University Animal Research Committee, following the Guide for the Care and Use of Laboratory Animals (NIH publication no. 85–23, revised 1996).

After a week of adaptive feeding, 12 mice were randomly selected as the control group. Except for the control group, the other animals were injected intraperitoneally with D-galactose, 100 mg/kg body weight, at a dose of 0.1 mL/10 g for 6 weeks. The control group was injected intraperitoneally with the same amount of normal saline once a day for 6 weeks. After 6 weeks, the blood was collected from the tail vein, and the serum was separated at a speed of 3000 rpm/10 min. The content of MDA in the serum was determined, and the patients were randomly divided into two groups, according to the level of MDA. After grouping, there was no significant difference in the MDA level among the model groups (*p* > 0.05), but there was significant difference in MDA level between the model group and the control group (*p* < 0.05).

After the model was established, they were randomly divided into one model control group, one whey protein group (1.5 g/kg body weight), and three whey protein peptide intervention groups (0.3 g/kg body weight, 1.5 g/kg body weight, 3.0 g/kg body weight). The test samples were administered by gavage every day, the control group and model control group were given high-pressure sterilized distilled water, the whey protein group and whey protein peptide group were given corresponding concentrations of test substance; whey protein peptide and whey protein solution were prepared with distilled water. While the test samples were given, the model control group, whey protein group, and each dose group were given the same dose of D-galactose intraperitoneally, at the same time, the control group was administered intraperitoneal injections of normal saline. The animals were weighed twice a week, and the sample dose was adjusted according to body weight. The test samples were given for 30 days. During the experiment, the general conditions of mice in each group were observed weekly, including hair color, mental state, food intake, and daily activities.

### 2.4. Open-Field Test

Wooden box, length × width × height: 45 cm × 45 cm × 45 cm, lined with black pad paper, with a white bottom, and 5 × 5 square grid. During the experiment, the mice were gently put into the central grid, and the following was recorded: number of squares crossed, the number of squares crossed rearing, the times spent in inner squares, the number of inner squares entered, and the number of fecal boli.

### 2.5. Morris Water Maze Test

During the experiment, the platform was randomly placed in the first quadrant and fixed 1~1.5 cm underwater, and the water temperature was maintained at 21 °C ± 1 °C. The whole experiment process was divided into two parts: place navigation test and spatial probe test.

Place navigation test: the experiment lasted for 6 days. During the test, the platform was placed in the center of the first quadrant of the round pool and fixed. Every day, the mice were gently put into the water from the east, west, south, and north quadrants facing the pool wall. The time (escape latency), swimming distance, and average swimming velocity of the mice from entering the water to finding the platform were recorded. Each test interval was 5 min, and the average score (of the four times) was taken as the final score of the day (to enter the final statistics). If the animal did not find the platform within 60 s, the experimenter guided the animal to the platform, stayed on the platform for 10 s, and the escape latency was recorded as 60 s.

Spatial probe test: the method was to remove the platform on the 7th day and put the mice into the water from any entry point in the third quadrant. The time from entering the water to the first arrival of the platform was recorded as the escape latency. The residence time of the mice in the target quadrant (the quadrant where the platform was in the navigation experiment), the swimming distance in the target quadrant, and the number of times of accurately crossing the platform in 60 s were recorded.

### 2.6. Novel Object Recognition Test

The new object recognition experiment was carried out in an open field experimental device (40 cm × 40 cm × 40 cm). There were three objects: A, B, and C. Object A and B were exactly the same, while object C was completely different from object A and B, all objects were heavy enough to prevent mice from pushing. The experiment was divided into three steps: adaptation period, training period, and test period. The first day was the adaptation period: mice were placed in the middle of the test box, without any objects in the box, and they were adaptive for 5 min. The second day was the training period and the detection period: during the training period, two identical toys (A and B) were placed in the experimental device, 10 cm away from the two sides of the wall. The mice were placed with their backs toward the object, and at the same distance from the object from the box. They were familiar with the objects for 5 min, and the exploration situation between the mice and the two objects was recorded. Test period: after 5 h of rest, we replaced one of the objects (B with toy C) and put it in the box (A and C) in the same position. We placed the mouse back to the object, at the same distance from the object in the box, and observed for 5 min. The mice and the two objects were recorded. Based on the number of times the mouse’s nose or mouth touched the object, and the exploration time within 2 cm from the object, the front paw on the object, sniffing, licking, biting, etc., were all exploration objects, but climbing, or climbing objects, were not exploration objects. We used the following formula to calculate the new object index (NOI): new object recognition index = time to explore new objects/(time to explore new objects + time to explore old objects) × 100%.

### 2.7. Hematoxylin and Eosin (HE) Staining of Hippocampus

At the end of the experiment, the mice were killed by cervical dislocation. Immediately after the animals were killed, the brain tissue was separated from the ice and fixed with 4% paraformaldehyde solution for more than 48 h. The part containing the hippocampus was cut from the coronal plane and paraffin embedded for serial coronal sections. We performed HE staining of the slices, dehydrated and mounted the slides, used a microscope to check the images, and collected and analyze the images.

### 2.8. TUNEL Labeling of Hippocampus CA1 Region

After the experiment, the mice were killed by cervical dislocation. The animals were craniotized immediately, and the brain tissues were separated on ice, and fixed with 4% paraformaldehyde solution for more than 48 h. The coronal surface was cut from the hippocampus, and serial coronal sections were embedded in paraffin. We strictly followed the instructions of the TUNEL fluorescent staining kit. Quantitative analysis: we used CaseViewer C.V 2.3 to observe the slice results at 40x magnification. Each mouse randomly selected four non-overlapping fields in the hippocampal CA1 area to count positive apoptotic cells.

### 2.9. Biochemical Assays and Enzyme-Linked Immunosorbent Assay

At the end of the experiment, the mice were killed by eyeball blood sampling and cervical dislocation. After the animals were killed, the liver and brain were separated on ice immediately, and the excess tissue was removed for standby. The levels of serum, liver, and brain oxidative stress biomarkers (SOD, GSH-Px, MDA, PC) were determined by assay kits, according to the protocol provided by the manufacturer. The brain tissue homogenate of different groups was used to estimate the inflammatory parameters (TNF-α, IL-1β), cholinergic system related enzymes (AChE, ChAT) by assay, according to the protocol provided by the manufacturer.

### 2.10. Western Blot Analysis

The expression of p-CREB, CREB, p-CaMKⅡ, CaMKⅡ, and BDNF proteins in brain tissue of the experiment mouse was determined by Western blot analysis. At the end of the experiment, the mice were killed by cervical dislocation. Immediately after the animals were killed, the brain tissue was separated on ice. The fresh brain tissue was about 20 mg, and 200 μL protein lysate was added to every 10 mg tissue. It was homogenized on ice with a glass grinder. The homogenate was transferred to a pre-cooled 1.5 mL EP tube and placed on ice for 15 min to fully crack (4 °C, 12,000 rpm for 10 min). The supernatant, after centrifugation, was transferred into a 0.5 mL centrifuge tube and frozen at −20 °C. Then, we followed the sequence of electrophoresis, transfer, blocking, primary antibody incubation, secondary antibody incubation, color development, and exposure, using the Western Blot method to detect the hippocampus; the surrounding cortex p-CREB, CREB, p-CaMKⅡ, CaMKⅡ, and BDNF expression level was determined.

### 2.11. Statistical Analysis

Statistical analyses were performed using SPSS software version 24 (SPSS Inc., Chicago, IL, USA). All values were presented as mean ± standard deviation (SD). Differences between groups were analyzed by a one-way analysis of variance test and LSD methods if the data were homogeneous, or the Tamhane T3 test if variances were unequal. A value of *p* < 0.05 was considered statistically significant.

## 3. Results

### 3.1. Effects of WHP on the Space Exploration Capability of the Open-Field Test

In the open-field test, the number of squares crossed and rearing of the model control group were significantly reduced, compared with the control group (*p* < 0.05). Compared with the model control group, the number of squares crossed in WHP-MG (1.5 g/kg body weight) and WHP-HG (3.0 g/kg body weight) significantly increased (*p* < 0.05). Moreover, the number of rearing in the whey protein group, WHP-LG (0.3 g/kg body weight), WHP-MG (1.5 g/kg body weight), and WHP-HG (3.0 g/kg body weight) groups were significantly higher than those in the model control group (*p* < 0.05). However, there was no significant difference in the times spent in the inner squares, the number of inner squares entered, and the number of fecal boli between groups (*p* > 0.05) (Table 2).

### 3.2. Effects of WHP on the Learning and Memory Capability of the Morris Water Maze

During the place navigation test, with the increase of training days, the duration of escape latency in each group showed a decreasing trend. On day 6, compared with day 1, the escape latency of mice in the control group, whey protein group, and WHP-LG, WHP-MG, WHP-HG groups was significantly shortened (*p* < 0.05). However, there was no significant difference in the escape latency between the model control group on day 6 and day 1 (*p* > 0.05). In terms of the average swimming velocity, the model control group was significantly decreased compared with the control group on the first, third, and sixth day (*p* < 0.05). The swimming velocity of the whey protein group was significantly higher than that of the model control group on the first day of the experiment (*p* < 0.05), while the swimming velocity of the WHP-HG group was significantly different from that of the whey protein group (*p* < 0.05). While, no significant difference was found in the swimming distance of each group (*p* > 0.05) (Table 3).

During the spatial probe test, the escape latency in the model group was significantly more than that in the control group. Moreover, the numbers in regards to the platform crossed, time spent in the target quadrant, and distance in target quadrant in the model group were significantly less than those in the control group (*p* < 0.05). Compared with the model control group, the distance in the target quadrant in the whey protein group was significantly longer (*p* < 0.05). Meanwhile, the escape latency in the WHP-HG group was significantly shortened (*p* < 0.05), the time spent in the target quadrant and distance in the target quadrant in the WHP-HG group was significantly higher than in the model control group (*p* < 0.05) (Table 4).

**Figure 1:**
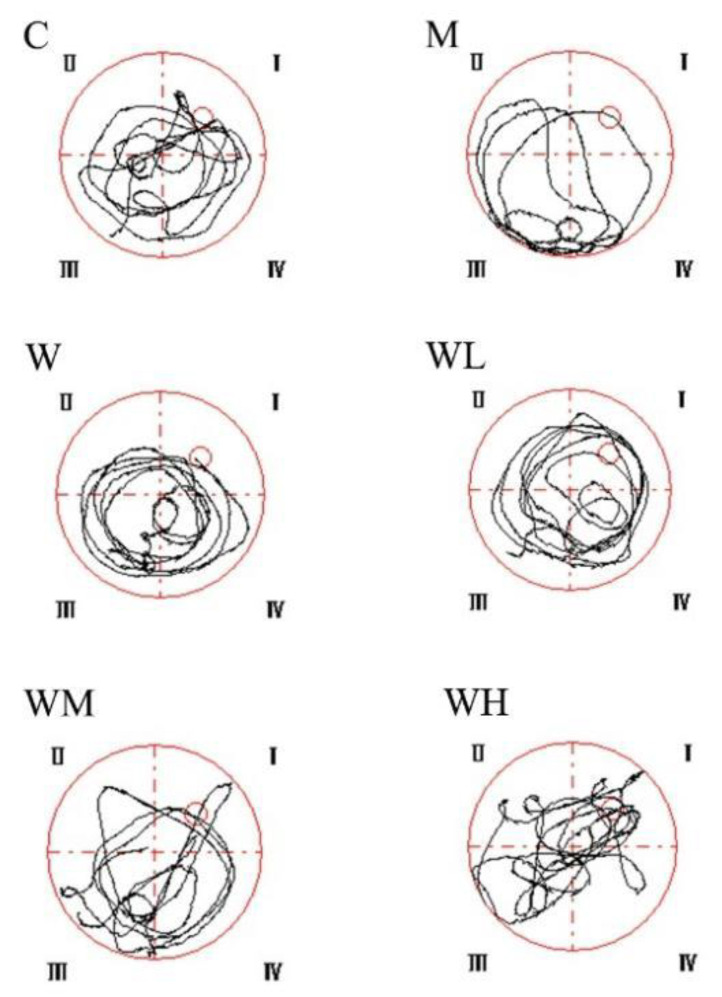
Typical roadmap of mice in the spatial probe test. C, control group; M, model control group; W, whey protein group at a dose of 1.5 g/kg; WL, whey protein peptide low dose group at a dose of 0.3 g/kg; WM whey protein peptide medium dose group at a dose of 1.5 g/kg; WH, whey protein peptide high dose group at a dose of 3.0 g/kg.

The typical roadmap of mice in each group in the spatial probe test is shown in Figure 1. The mice in the control group, WHP-MG, and WHP-HG groups occupy a relatively high proportion of movement in the target quadrant where the platform is located, meanwhile, the retention time of those groups are longer. Mice in the model control group swim less around the platform and through the target quadrant.

### 3.3. Effects of WHP on the Learning and Memory Capability of the Novel Object Recognition

**Figure 2:**
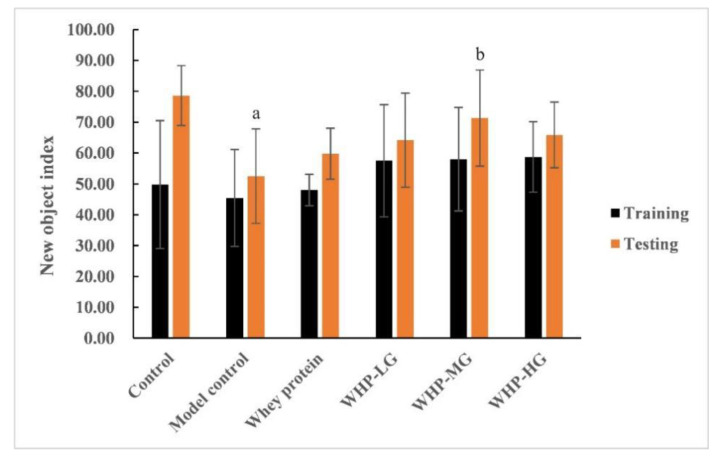
Effects of WHP on the learning and memory capability of the novel object recognition. Data are expressed as means ± SD (*n* = 8). ^a^ *p* < 0.05 indicates significant difference versus the control group; ^b^ *p* < 0.05 indicates significant difference versus the model group. Whey protein group at a dose of 1.5 g/kg; WHP-LG, whey protein peptide low dose group at a dose of 0.3 g/kg; WHP-MG, whey protein peptide medium dose group at a dose of 1.5 g/kg; WHP-HG, whey protein peptide high dose group at a dose of 3.0 g/kg.

During the novel object recognition test, there was no significant difference in the new object index (NOI) of each group during training (*p >* 0.05). However, NOI of the model control group was significantly lower than that of the control group during testing (*p* < 0.05). Compared with the model control group, NOI of the WHP-MG group was significantly higher in testing (*p* < 0.05) (Figure 2).

### 3.4. Effects of WHP on the Hematoxylin and Eosin (HE) Staining of Hippocampus

**Figure 3:**
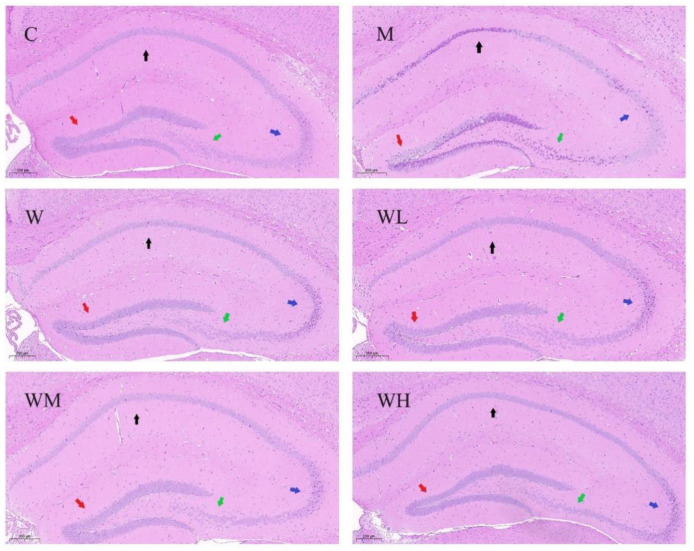
HE staining of the coronal section of the hippocampus in each group. C, control group; M, model control group; W, whey protein group at a dose of 1.5 g/kg; WL, WHP-LG, whey protein peptide low dose group at a dose of 0.3 g/kg; WM, WHP-MG, whey protein peptide medium dose group at a dose of 1.5 g/kg; WH, WHP-HG, whey protein peptide high dose group at a dose of 3.0 g/kg.

As shown in Figure 3, in the control group, the cells of the hippocampus in each region were arranged neatly, with full and normal structural morphology, clear membrane boundary, and no obvious pathological changes. Compared with the control group, apoptosis occurred in all regions of the model control group, with irregular cell arrangement, unclear cell membrane boundary, and darker staining. Compared with the model control group, the apoptosis of cells in the whey protein group was slightly better, but there were still some cells with deep staining and irregular cell shape changes. The apoptosis of cells in each region of the WHP dose group was better, and the staining color was lighter, and there was less deformation and disorder of the cell membrane, but the improvement of the hippocampal nerve cells in the CA2 region was less. Among them, the pathological characteristics of hippocampal neurons in WHP-MG improved the best, which was closest to the HE staining of the control group.

**Figure 4:**
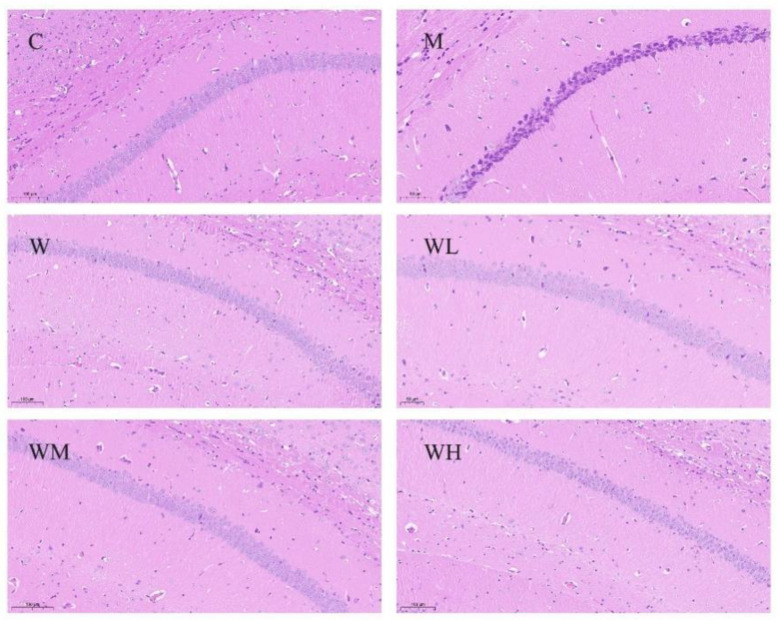
HE staining of the hippocampal CA1 region in each group. C, control group; M, model control group; W, whey protein group at a dose of 1.5 g/kg; WL, WHP-LG, whey protein peptide low dose group at a dose of 0.3 g/kg; WM, WHP-MG, whey protein peptide medium dose group at a dose of 1.5 g/kg; WH, WHP-HG, whey protein peptide high dose group at a dose of 3.0 g/kg.

As shown in Figure 4, the cells in the hippocampal CA1 region of mice in the control group were regular in morphology, orderly in arrangement, clear in structure, uniform in nucleus staining, and no obvious apoptotic cells were observed. However, the CA1 cells in the model control group showed obvious pathological morphological changes of apoptosis, a large number of cells were arranged irregularly, the cell membrane edge was blurred, the nucleus and cytoplasm boundaries were not clear, the cell morphology was irregular and deeply stained. Meanwhile, compared with the model control group, the cell morphology of the CA1 region of mice in the whey protein group and WHP dose groups improved to varying degrees, and the number of apoptotic cells was reduced. Among them, the morphology of hippocampal neurons in the WHP-MG and WHP-HG was the most similar to the control group.

### 3.5. Effects of WHP on the TUNEL Labeling of Hippocampus CA1 Region

**Figure 5:**
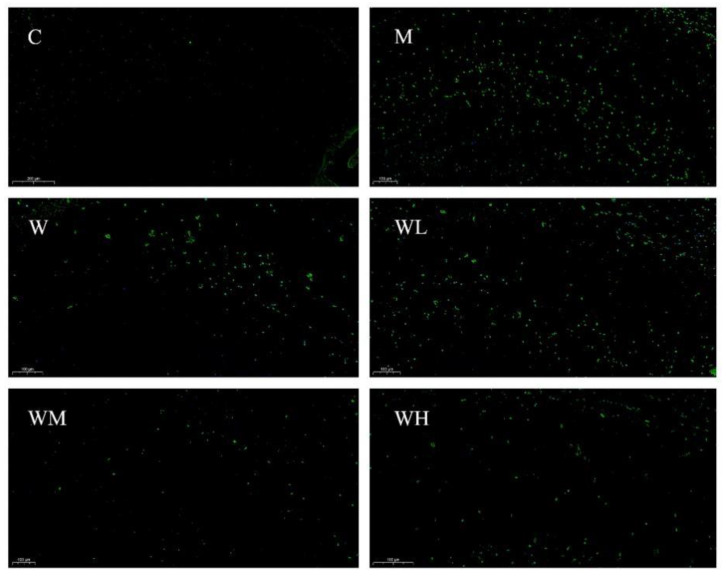
TUNEL labeling of the hippocampal CA1 region in each group. C, control group; M, model control group; W, whey protein group at a dose of 1.5 g/kg; WL, WHP-LG, whey protein peptide low dose group at a dose of 0.3 g/kg; WM, WHP-MG, whey protein peptide medium dose group at a dose of 1.5 g/kg; WH, WHP-HG, whey protein peptide high dose group at a dose of 3.0 g/kg.

**Figure 6:**
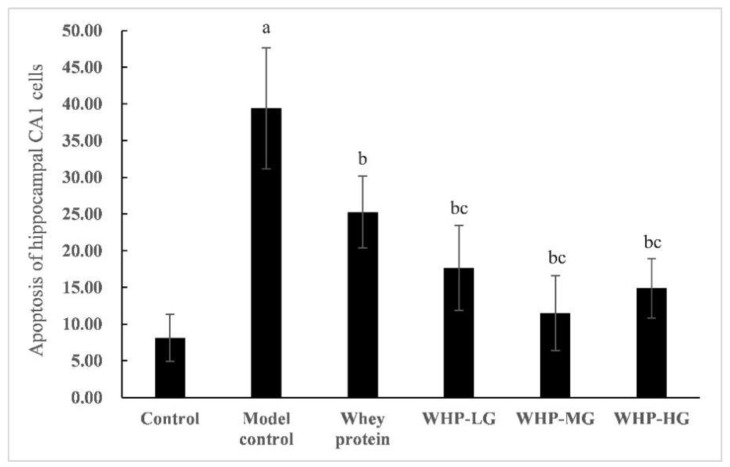
Effects of WHP on the TUNEL labeling of the hippocampus CA1 region. Data are expressed as means ± SD (*n* = 8). ^a^ *p* < 0.05 indicates significant difference versus the control group; ^b^ *p* < 0.05 indicates significant difference versus the model group; ^c^ *p* < 0.05 indicates significant difference versus the whey protein group. Whey protein group at a dose of 1.5 g/kg; WHP-LG, whey protein peptide low dose group at a dose of 0.3 g/kg; WHP-MG, whey protein peptide medium dose group at a dose of 1.5 g/kg; WHP-HG, whey protein peptide high dose group at a dose of 3.0 g/kg.

As shown in Figure 5 and Figure 6, TUNEL labeling under fluorescence microscope showed that, compared with the control group, the number of apoptosis cells in the model control group significantly increased (*p* < 0.05). Compared with the model control group, the number of TUNEL positive cells in the whey protein group and WHP dose groups significantly decreased (*p* < 0.05). Moreover, the number of apoptotic cells in WHP dose groups decreased more than whey protein group (*p* < 0.05).

### 3.6. Effects of WHP on the Aging-Induced Oxidative Stress of Mice

**Figure 7:**
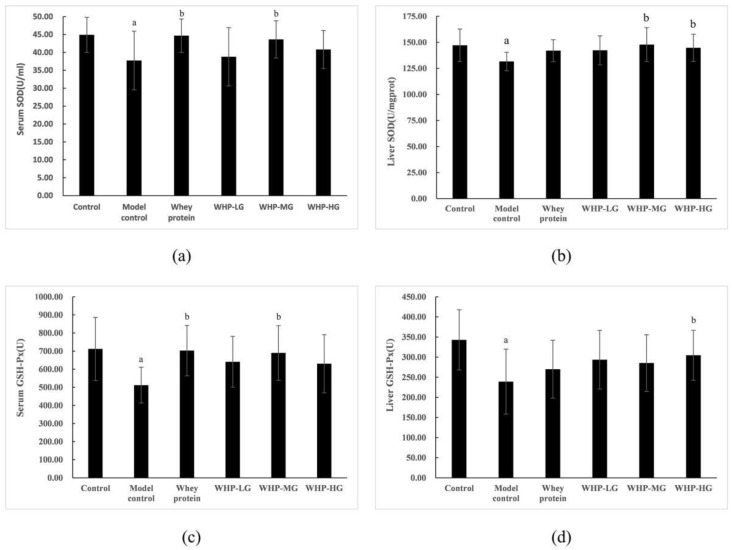
Effects of WHP on serum SOD (**a**), liver SOD (**b**), serum GSH-Px (**c**), liver GSH-Px (**d**). Data are expressed as means ± SD (*n* = 12). ^a^ *p* < 0.05 indicates significant difference versus the control group; ^b^ *p* < 0.05 indicates significant difference versus the model group. SOD, superoxide dismutase; GSH-Px, glutathione peroxidase. Whey protein group at a dose of 1.5 g/kg; WHP-LG, whey protein peptide low dose group at a dose of 0.3 g/kg; WHP-MG, whey protein peptide medium dose group at a dose of 1.5 g/kg; WHP-HG, whey protein peptide high dose group at a dose of 3.0 g/kg.

As shown in Figure 7a,b, SOD activity in the serum and liver of the model control group significantly decreased compared to those in the control group (*p* < 0.05). Compared with the model control group, serum SOD activity in the whey protein group significantly increased (*p* < 0.05); serum, and liver SOD activity in WHP-MG and WHP-HG significantly increased (*p* < 0.05).

Meanwhile, there was significantly differences in the activity of GSH-Px in the serum and liver, between the model control group and the control group (*p* < 0.05). Compared with the model control group, serum GSH-Px activity in the whey protein group significantly increased (*p* < 0.05); moreover, serum and liver GSH-Px activity in WHP-MG and WHP-HG respectively increased (*p* < 0.05) (Figure 7c,d).

**Figure 8:**
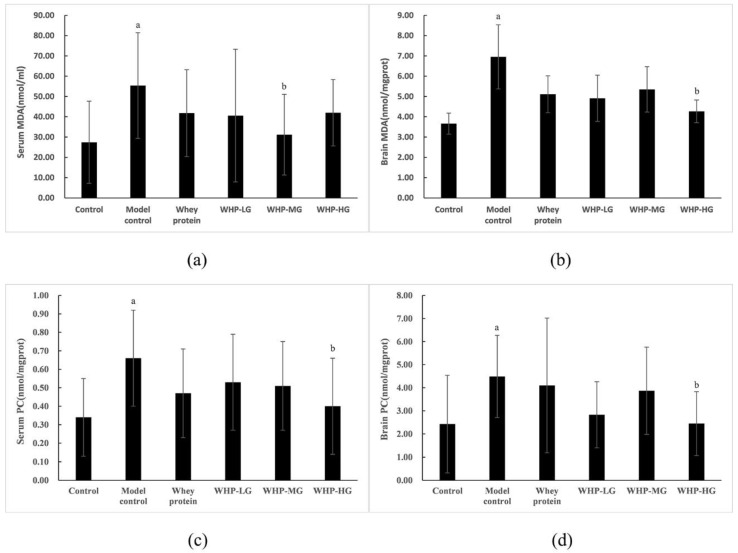
Effects of WHP on serum MDA (**a**), brain MDA (**b**), serum PC (**c**), brain PC (**d**). Data are expressed as means ± SD (*n* = 12). ^a^ *p* < 0.05 indicates significant difference versus the control group; ^b^ *p* < 0.05 indicates significant difference versus the model group. MDA, malondialdehyde; PC, protein carbonyl. Whey protein group at a dose of 1.5 g/kg; WHP-LG, whey protein peptide low dose group at a dose of 0.3 g/kg; WHP-MG, whey protein peptide medium dose group at a dose of 1.5 g/kg; WHP-HG, whey protein peptide high dose group at a dose of 3.0 g/kg.

As shown in Figure 8a,b, MDA content in serum and brain tissue of the model control group was markedly higher compared to the mice in the control group (*p* < 0.05). Compared with the model control group, the serum MDA content of mice in WHP-MG was lower (*p* < 0.05), while the brain MDA content of mice in WHP-HG greatly decreased (*p* < 0.05).

As shown in Figure 8c,d, the content of protein carbonyl in serum and brain tissue of the model control group was significantly higher than that of the control group (*p* < 0.05). In comparison with the model control group, the levels of PC in serum and brain tissue of WHP-HG significantly decreased (*p* < 0.05).

### 3.7. Effects of WHP on the AChE and ChAT Activity in the Brain Tissue of Mice

**Figure 9:**
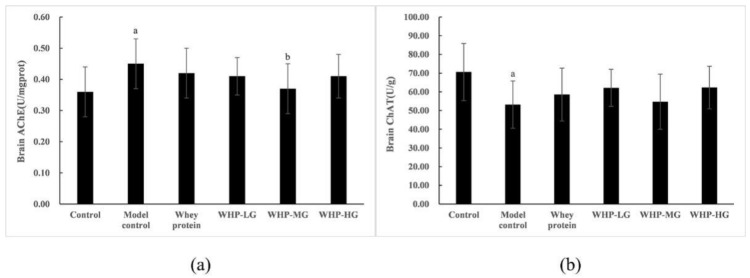
Effects of WHP on brain AChE (**a**) and brain ChAT (**b**). Data are expressed as means ± SD (*n* = 8). ^a^ *p* < 0.05 indicates significant difference versus the control group; ^b^ *p* < 0.05 indicates significant difference versus the model group. AChE, acetyl cholinesterase; ChAT, choline acetyltransferase. Whey protein group at a dose of 1.5 g/kg; WHP-LG, whey protein peptide low dose group at a dose of 0.3 g/kg; WHP-MG, whey protein peptide medium dose group at a dose of 1.5 g/kg; WHP-HG, whey protein peptide high dose group at a dose of 3.0 g/kg.

As shown in Figure 9a,b, a marked difference was found in AChE and ChAT activity in the brain tissue between the control group and the model control group (*p* < 0.05). The treatment (medium dose) of WHP significantly increased the AChE activity of aging mice (*p* < 0.05). However, the ChAT activity of the whey protein group, WHP-LG, WHP-MG, WHP-HG showed no great difference (*p* > 0.05).

### 3.8. Effects of WHP on the Level of Proinflammatory Factors in the Brain Tissue of Mice

**Figure 10:**
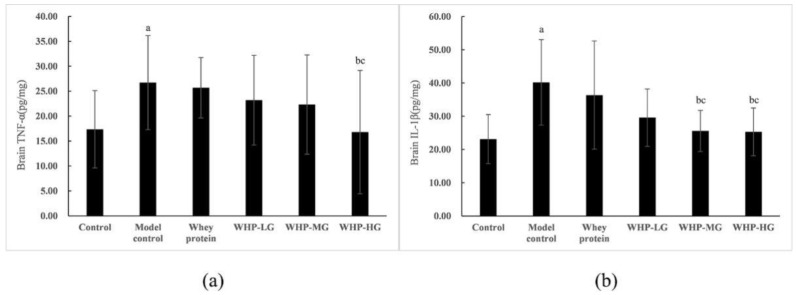
Effects of WHP on brain TNF-α (**a**), brain IL-1β (**b**). Data are expressed as means ± SD (*n* = 8). ^a^ *p* < 0.05 indicates significant difference versus the control group; ^b^ *p* < 0.05 indicates significant difference versus the model group; ^c^ *p* < 0.05 indicates significant difference versus the whey protein group. TNF-α, tumor necrosis factor-α; IL-1β, interleukin-1β. Whey protein group at a dose of 1.5 g/kg; WHP-LG, whey protein peptide low dose group at a dose of 0.3 g/kg; WHP-MG, whey protein peptide medium dose group at a dose of 1.5 g/kg; WHP-HG, whey protein peptide high dose group at a dose of 3.0 g/kg.

As shown in Figure 10a,b, compared with the control group, the concentrations of IL-1β and TNF-α in brain tissue of the model control group were significantly increased (*p* < 0.05). After intervention of WHP, WHP-MG showed lower expression levels of IL-1β than the model control group, while WHP-HG showed a great decrease of IL-1β and TNF-α in brain tissue (*p* < 0.05).

### 3.9. Effects of WHP on the Expression of Synaptic Plasticity Related Proteins in the Brain Tissue of Mice

**Figure 11:**
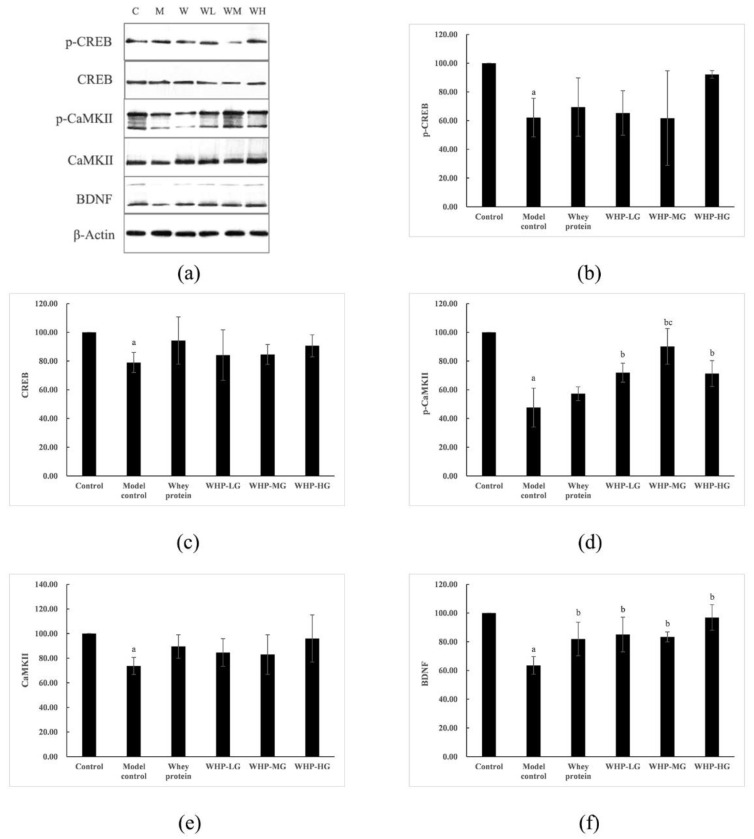
Effects of WHP on the expression of synaptic plasticity related proteins in the brain tissue of mice. (**a**) Synaptic plasticity related proteins were analyzed by Western blot. C, control group; M, model control group; W, whey protein group at a dose of 1.5 g/kg; WL, WHP-LG, whey protein peptide low dose group at a dose of 0.3 g/kg; WM, WHP-MG, whey protein peptide medium dose group at a dose of 1.5 g/kg; WH, WHP-HG, whey protein peptide high dose group at a dose of 3.0 g/kg. (**b**) Effects of WHP on p-CREB in the brain tissue of mice. (**c**) Effects of WHP on CREB in the brain tissue of mice. (**d**) Effects of WHP on p-CaMKⅡ in the brain tissue of mice. (**e**) Effects of WHP on CaMKⅡ in the brain tissue of mice. (**f**) Effects of WHP on BDNF in the brain tissue of mice. Data are expressed as means ± SD (*n* = 8). ^a^ *p* < 0.05 indicates significant difference versus the control group; ^b^ *p* < 0.05 indicates significant difference versus the model group; ^c^ *p* < 0.05 indicates significant difference versus the whey protein group. CREB, cAMP-response element binding protein; CaMKⅡ, calcium/calmodulin-dependent protein kinases Ⅱ; BDNF, brain-derived neurotrophic factor. Whey protein group at a dose of 1.5 g/kg; WHP-LG, whey protein peptide low dose group at a dose of 0.3 g/kg; WHP-MG, whey protein peptide medium dose group at a dose of 1.5 g/kg; WHP-HG, whey protein peptide high dose group at a dose of 3.0 g/kg.

As shown in Figure 11, the contents of p-CREB, CREB, p-CaMKⅡ, CaMKⅡ, and BDNF in the brain tissue of the model control group significantly decreased (*p* < 0.05). The expression of p-CaMKⅡ in WHP-LG, WHP-MG, WHP-HG, compared with the model control group, showed a great increase (*p* < 0.05). Moreover, the improvement of WHP-MG was better than that of the whey protein group (*p* < 0.05); the level of BDNF in the brain tissue of the whey protein group and all WHP groups was markedly higher than the model control group (*p* < 0.05). However, no statistical difference was observed among the contents of p-CREB, CREB, and CaMKⅡ between the groups (*p* > 0.05).

## 4. Discussion

The decline of physiological and biochemical functions associated with aging is inevitable for every individual. Aging will lead to the accumulation of oxidative stress and damage to brain functioning, to varying degrees, typically manifested as learning and memory dysfunction. Whey protein has a variety of active components, such as β-lactoglobulin, α-lactalbumin, immunoglobulin, glyco-giant peptide protein, serum albumin, lactoferrin, milk peroxidase, etc. It is proven to have a variety of biological activities, such as antioxidant, antimicrobial, and blood glucose regulation [5,6,7]. The WHP isolated from whey protein by enzymatic hydrolysis has also been demonstrated to have a variety of biological activities. The present study shows that WHP can significantly improve the aging-related decline of exploration and exercise ability, and promote spatial and non-spatial learning and memory. Specific mechanisms include reducing the hippocampal nerve cell degeneration, reducing nerve cell apoptosis, improving cholinergic function, reducing the brain tissue inflammatory factor expression, reducing oxidative stress damage, reducing β-amyloid protein deposition, and improving p-CaMKⅡ and BDNF processes. WHP has the potential to improve aging-related learning/memory impairment.

In this study, the aging model was established by intraperitoneal injection of D-galactose, and the intervention effect of WHP on aging-related cognitive impairment was observed. Excessive intake of D-galactose will reduce to galactitol under the action of aldose reductase; however, galactitol cannot be metabolized normally. The accumulation of galactitol will induce the increase of cell osmotic pressure and the variation of cell volume, which will lead to related dysfunction and metabolic disorders. In addition, studies have confirmed that long-term D-galactose intake can activate and promote the formation of ROS, such as H_2_O_2_. Active oxygen free radicals have a strong affinity for proteins, lipids, and other macromolecules. D-galactose can trigger the accumulation of advanced glycation end products (AGEs) and promote its binding with the receptor of advanced glycation end products [8]. It can reduce the activity of antioxidant enzymes, and long-term intake will cause lipid peroxidation and protein carbonyl accumulation, promote the formation of oxidative stress, and accelerate the aging process. It has been reported that excessive supply of D-galactose in rodents not only results in abnormal metabolism of organs and tissues caused by accumulation of ROS in viscera and circulation, but also causes neurotoxicity to brain, and induces decline of learning, memory ability, and cognitive impairment, the mechanisms of which are related to oxidative stress injury, neuroinflammation, cholinergic dysfunction, neurogenesis decreases, and neuronal apoptosis [9]. Excessive intake of D-galactose can cause the decline of the endogenous antioxidant defense mechanism and the oxidative damage of macromolecules. In brain tissue, the accumulation of lipid peroxide and protein carbonyl products can accelerate brain aging and induce brain function damage [10]. The aging model constructed by D-galactose can simulate the learning, memory, and cognitive impairment of the elderly with aging. In recent years, it was widely used in the study of age-dependent neurodegenerative diseases, such as Alzheimer’s disease [11]. The aging model constructed by D-galactose has similar physiological and biochemical characteristics with natural aging. The literature shows that the aging degree simulated by the D-galactose model is close to that of normal aging mice, aged 16–24 months [12].

With the increase of age, people usually have different degrees of brain aging, which is typically manifested as an impairment in learning and memory functions. In this study, a variety of behavioral experiments was used to evaluate the learning and memory function of the D-galactose aging mice models after WHP intervention. The open field test is the best behavioral test to measure locomotor activity and anxiety-like behavioral responses at the same time [13]. Morris water maze is an experimental method that was designed by the British psychologist Morris in the early 1980s, and applies to this research in regards to the mechanisms of brain spatial learning and memory. It can be used to test spatial learning, orientation, and the response and positioning ability of animals. In recent years, it was widely used in senescence-related experiments. The new object recognition experiment is a precise and sensitive behavioral detection method to detect the non-spatial memory ability of mice by recording the exploration time of objects. According to the natural tendency of rodents to explore new stimuli and the location of stimuli, the learning and memory ability of new and old object positions can be detected [14]. The three behavioral results showed that the D-galactose aging mice models showed different degrees of motor, exploration, learning, and memory impairment in the open field test, Morris water maze test, and new object recognition test. In the open field experiment of the aging mice models, the number of squares crossed and rearing decreased. In the water maze experiment, the swimming speed decreased, the escape latency did not significantly improve after 6 d of training during the place navigation test. In the spatial probe test, the escape latency was greatly extended, the number of platform crossings, the target quadrant residence time, and the target quadrant distance of aging mice were shortened. Moreover, the NOI index of the model group decreased in the new object recognition experiment. However, studies have shown that medium (1.5 g/kg body weight) and high (3.0 g/kg body weight) doses of WHP can significantly improve the number of squares crossed and standing times, and improve the age-related spatial exploration dysfunction in mice in open field experiments. In the Morris water maze experiment, the escape latency of mice in each WHP dose group significantly improved in the positioning navigation test after 6 d of training, suggesting that WHP has the potential to enhance learning and memory ability. In space exploration experiments, high dose (3.0 g/kg body weight) WHP intervention markedly improved the prolonged escape latency induced by intraperitoneal injection of D-galactose, and significantly increased the time spent in the target quadrant and the distance in the target quadrant of mice during the experiment. In the NOR experiment, the medium dose (1.5 g/kg body weight) of WHP increased the exploration time of new objects during the detection period and enhanced the cognitive non-spatial learning and memory ability of mice. In conclusion, the results of the three behavioral experiments suggest that WHP can improve age-related learning and memory disorders.

The hippocampus, which is located in the medial temporal region of the brain, and is considered related to information storage and retrieval, is the most frequently discussed structure in the studies related to brain aging, learning and memory, and cognitive impairment. The hippocampus has a unique shape and cellular structure, which is composed of CA1, CA2, CA3 pyramidal cell layer, and dentate gyrus DG. The structures of each part are interrelated to process the sensory and memory information input from the cerebral cortex. The hippocampus has a unidirectional three-synaptic pathway, originating from the entorhinal cortex; it is projected to the DG region, CA3 subregion, and CA1 subregion, in turn. The operation of the DG region, combined with the CA1 and CA3 subregions, constitutes a complete circuit, and plays a vital role in the normal maintenance of the structure and function of the hippocampus [15]. Among them, the DG region is responsible for processing metric spatial representation; the CA3 subregion is responsible for spatial pattern association, new information detection, and short-term memory formation; the CA1 subregion mediates temporal pattern association and the process of medium-term memory [16]. The researchers believe that the appearance of age-related learning and memory disorders is closely related to the changes in the micromorphology of various regions of the hippocampus. First, aging is accompanied by a decrease in brain weight, which is partially related to the loss of hippocampal nerve cells and the occurrence of apoptosis. For example, the number of synapses in the DG region is widely reduced in the hippocampus of old animals [17,18]. Secondly, studies have found that people with mild cognitive impairment have extensive neuronal degeneration before the onset of brain atrophy. Studies have also shown that the CA1 subregion is the brain region that initially shows pathological state and neuron loss in Alzheimer’s disease patients [19,20]. In this study, the results of HE staining and the TUNEL assay showed that the D-galactose aging mice models had extensive neurodegeneration in various regions of the hippocampus and changes in the CA1 subregion neuronal apoptosis, suggesting the loss of hippocampal neurons during the aging process. The intervention of whey protein and WHP in each dose group could significantly improve the degeneration of neurons in the hippocampal DG and CA1 region, maintain the morphology of neurons, and significantly reduce the number of apoptosis cells in the hippocampal CA1 region of aging mice. Among them, the low (0.3 g/kg body weight), medium (1.5 g/kg body weight), and high (3.0 g/kg body weight) WHP dose groups improved the CA1 region cell apoptosis greatly—better than the whey protein group. Combined with the results of the behavioral experiments, the decline of learning and memory function of aging individuals may be closely related to the destruction of hippocampal neuronal microenvironment homeostatic. The intervention of WHP can alleviate the degeneration, necrosis, and apoptosis of neurons in the aging process, and maintain the physiological infrastructure of normal brain functions. Therefore, it plays a key role in improving age-related cognitive impairment and delaying the decline of learning and memory function.

A number of studies have shown that oxidative stress damage is closely related to aging-related learning/memory impairment. The endogenous ways of resisting oxidative stress and aging are mostly achieved through antioxidant enzymes SOD and GSH-Px. The oxygen consumption of brain tissue can be as high as 20% of the whole body, and it has a low level of endogenous defense mechanisms of antioxidant enzymes. Therefore, the brain is more vulnerable to a ROS attack and damage, which makes the increase and accumulation of oxidative stress caused by aging closely related to the high incidence of age-dependent neurodegenerative diseases [21]. MDA is an important indicator to measure the level of lipid peroxidation, and it is a common indicator to evaluate the degree of aging. Excessive accumulation of MDA in brain tissue can interfere with cell signal transduction, destroy membrane binding protein, lead to abnormal gene expression and energy metabolism, and cause irreversible damage. Protein carbonylation is the direct product of protein damage induced by free radicals, and direct protein carbonylation may lead to the loss of protein function. In this study, compared with the control group, the levels of antioxidant enzymes in the serum and liver of the aging mice models decreased, which was consistent with the theory of free radicals of aging. SOD and GSH-PX are important antioxidants in vivo, and their activities are closely related to the level of oxidative stress. Through intervention, a medium dose (1.5 g/kg body weight) of WHP can significantly improve the activities of SOD in the serum, and GSH-PX in the serum and liver of aging mice. A high dose (3.0 g/kg body weight) of WHP interfering with the decline of activities of SOD and GSH-PX is reflected in the liver. The results show that WHP can enhance the endogenous antioxidant defense level of aging individuals, protect the body from oxidative stress damage, and play a role in reducing the dysfunction of various organs and tissues caused by ROS damage. Moreover, in this study, the serum and brain tissue of the aging mice models showed high accumulation of MDA and PC, which showed the oxidative stress injury related to brain aging. Under the intervention of WHP, compared with the model control group, the high dose (3.0 g/kg body weight) group showed a decrease of the MDA accumulation level in the brain tissue and PC level in the brain tissue, indicating that WHP can improve the oxidative stress level in the serum and brain tissue of aging mice. WHP can repair the oxidative defense mechanism of aging individuals, reduce the accumulation of lipid peroxidation and protein peroxidation, slow down brain aging, maintain healthy brain function, and it has a good application prospect in the development of healthy aging and reducing the risk of neurodegenerative diseases.

Acetylcholine is a neurotransmitter that plays an important role in the process of learning and memory. A large number of studies have confirmed that the normal maintenance of hippocampal cholinergic system functioning can promote normal progress of memory-related activities. The cholinergic system plays an important role in regulating and inducing synaptic plasticity. Previous studies found that the brains of AD patients often show abnormal levels of AChE and ChAT, which induces the occurrence and development of cholinergic neuron loss and the degeneration of cholinergic fibers [22]. ChAT is an important promoter of cholinergic function, which can promote the synthesis of ACh by acetyl coenzyme A and choline. AChE can rapidly hydrolyze ACh in the protruding space to form acetate and choline. Each AChE molecule can hydrolyze 5000 ACh molecules per second, which is the key enzyme leading to the inactivation of acetylcholine in the body [23]. In this study, the aging mice with learning and memory dysfunction showed a great increase in AChE activity and a significant decrease in ChAT activity in brain tissue, indicating that there was obvious cholinergic dysfunction in aging mice. The results showed that a medium dose (1.5 g/kg body weight) of WHP could significantly improve the abnormal increase of AChE activity in mouse brain tissue. WHP can reduce the activity of AChE in brain tissue, slow down the rapid decomposition of ACh caused by aging, intervene in the disorder of synthesis and utilization of age-dependent acetylcholine, protect the normal operation of the cholinergic system in normal brain tissue, and play a role in preventing aging-related learning/memory impairment.

The chronic inflammatory state of the elderly is one of the risk factors for the high prevalence of various age-dependent chronic diseases. The same neuroinflammatory damage of the central nervous system has become an important cause of aging-related learning/memory impairment. Chronic inflammatory states can damage the normal functions of organs in the elderly and interfere with their regeneration, which is a sign of aging. There is low-level immune activation in aging brain tissue, characterized by a long-term increase of the expression levels of inflammatory factors, such as IL-1β and TNF–α [24,25]. TNF-α is an important pro-inflammatory cytokine. The increase of TNF-α levels in brain tissue can reduce the survival rate of newborn hippocampal neurons, lead to neurogenesis damage, and promote cell apoptosis. Hippocampus is an important part of the expression of IL-1β and its receptor in the brain. Studies have found that an excessive imbalance of IL-1β signal transduction can lead to the inhibition of the LTP process in the CA1 area of the hippocampus, affect the synaptic plasticity process, interfere with the protective effect of BDNF on the nervous system, damage the survival of neurons, and lead to the generation of hippocampus-dependent memory defects [26]. This study showed that, compared with the control group, there was an abnormal high expression of inflammatory factors IL-1β and TNF-α in the hippocampus and surrounding the cortex of the D-galactose aging mice models, which indicated that this study could better simulate the chronic inflammatory state of brain aging. After WHP intervention, the IL-1β concentration in the brain tissue of the aging mice models in the medium dose (1.5 g/kg body weight) and high dose (3.0 g/kg body weight) groups significantly reduced. Meanwhile, high dose (3.0 g/kg body weight) WHP also markedly improved the abnormal increase of TNF-α levels, and the level of inflammatory factor relief was significantly better than that of the whey protein group. Combined with the results of the behavioral experiments, WHP can alleviate the chronic inflammatory state of brain tissue of aging individuals by reducing the over expression of age-related inflammatory factors, and then reduce the occurrence of related inflammation-dependent hippocampal dysfunction. WHP has the potential to intervene in aging-related learning/memory impairment by improving the excessive inflammatory response of brain tissue.

The formation basis of learning and memory is related to the experience-dependent change of synaptic transmission efficiency (LTP and LTD), also called synaptic plasticity, and the synthesis of various proteins in the body is closely related to the formation of LTP. Aging is accompanied by transcriptional and epigenetic changes in brain tissue, which will eventually lead to abnormal expression of related proteins, and inhibit the LTP process, resulting in the decline of synaptic plasticity, influencing the learning and memory ability of the body. In the process of learning and memory, neurons are in an excited state. The activation of NMDAR will lead to the increase of intracellular Ca^2+^ concentration, and then trigger a series of calcium-dependent signal cascades. Among them, CaMKⅡ is considered a necessary condition to induce LTP formation and related plasticity of dendritic spines. It plays a key role in signal transmission between neurons, development of neural circuits, and cognitive function [27,28]. The results showed that spatial learning and memory functions of aging mice were impaired; the expression levels of CaMKⅡ and p- CaMKⅡ in the brain significantly decreased, which indicated that the damage of synaptic plasticity caused by abnormal Ca^2+^ regulation was closely related to age-dependent cognitive impairment. Under the intervention of WHP of low (0.3 g/kg body weight), medium (1.5 g/kg body weight), and high (3.0 g/kg body weight) dose groups, the expression level of Ca^2+^ regulation-related enzymes in the brain tissue of aging mice increased, in particular, the expression level of p-CaMKⅡ significantly improved. Among them, the expression level of p-CaMKⅡ in the medium (1.5 g/kg body weight) dose group was significantly higher than that in the whey protein group. CaMKⅡ and p-CaMKⅡ are the key enzymes of calcium regulation. The damage of calcium regulation homeostasis is an important reason threatening the LTP process of synaptic plasticity in the hippocampus of elderly individuals. WHP can alleviate the age-dependent abnormal calcium regulation and regulate the expression of synaptic plasticity-related proteins by intervening on the expression of CaMKⅡ and p-CaMKⅡ, to improve aging-related learning/memory dysfunction.

CREB is an important transcription factor for the maintenance of long-term synaptic plasticity. After phosphorylation, CREB can be activated by various enzymes, such as CaMK. Continuous phosphorylation of CREB (p-CREB) is considered necessary for enhancing LTP. It plays an important role in the control of neuronal function, such as participating in the regulation of neural differentiation and the transcription of genes related to learning and memory, assisting cell proliferation and survival [18,24,29]. BDNF is an important downstream target of CREB regulation. It is believed that BDNF mediates various neuronal processes in mammalian brain, including neuronal survival, differentiation and growth, synaptic formation, synaptic information transmission, neuronal plasticity, neural excitability, and cognitive function. In addition, BDNF affects the neurogenesis of dentate gyrus, including the structure and function of neural plasticity. High BDNF expression level help to protect neurons from infection or stimulation-induced damage [22,30]. Studies have shown that the expression of CREB-related signal in aging organisms is abnormal, and the level of BDNF will decrease with the increase of age. The persistent inflammatory reaction in aging organisms can reduce the expression of BDNF, which makes it a key mechanism for regulating aging-related neurological dysfunction [17,26]. In this study, the expression levels of CREB, p-CREB, and BDNF in the brain tissue of D-galactose mice models were significantly lower, which indicated that the model was established successfully and the CREB/BDNF signaling pathway was abnormal. Under the intervention of whey protein and WHP of each dose group, the level of BDNF in mouse brain tissue significantly increased. BDNF is a factor closely related to neurogenesis, differentiation, and nutrition. The increase of the BDNF expression level is a neuroprotective signal, and a specific manifestation of synaptic plasticity and LTP process enhancement. WHP can effectively alleviate the decline of the BDNF expression level caused by aging, suggesting that it has a potential neuroprotective effect, and can regulate synaptic plasticity by improving age-dependent BDNF deficiency. At present, the results of this study showed that there was no significant difference in the CREB expression level between each group and the model control group. Studies by Jennifer showed that enhancing the CREB protein level in the hippocampus is closely related to promoting the formation of long-term memory [31]. However, studies by Yu have shown that only changing CREB activity may not completely improve cognitive impairment related to aging [32]. At present, there are controversies concerning the relationship between CREB and the improvement of cognitive impairment related to aging. Therefore, in further research, we should focus on the mechanism of CREB regulating the treatment of aging-related learning/memory impairment.

Recently, whey protein peptide has become a popular research topic because of its unique structure and strong biological activity. At present, there is a preliminary understanding of its antioxidant functioning, in regards to improving learning and memory function. For example, WHP administration can enhance the antioxidant capacity of mice and reduce the level of oxidative stress [33]. WHP had the ability to improve the learning and memory function in the Y maze experiment, the new object recognition experiment, the platform jumping experiment, the dark avoiding experiment, and the water maze experiment in aging mice [34,35], the mechanisms of which may be related to antioxidation, downregulation of inflammatory markers, and upregulation of autophagy-related markers [36]. In this study, the methods and indicators used in the above studies were integrated to reach the same conclusion, in terms of antioxidant functions, and improvements in learning/memory abilities in animals in WHP behavioral experiments. In addition, this study provides a supplement to the mechanisms of WHP, in regards to improving the learning/memory abilities in elderly animals. The effects of WHP on the histopathological features of the hippocampal CA1 region in aging mice were studied by HE staining and the TUNEL assay for the first time. It was observed that WHP could interfere with the degeneration and apoptosis of neurons in the hippocampal CA1 region of mice; moreover, this study is the first to explore the effect of WHP on the expression of synaptic plasticity-related proteins in the brains of aging mice. Synaptic plasticity is considered the biological micro-basis of learning and memory. In this study, Western blot was used for the first time to analyze the effect of WHP on the expression levels of BDNF, CREB, CaMK, and other proteins in the brain of elderly mice. Positive results were obtained, providing a certain supplement for the mechanism of WHP in improving learning/memory function (and it had a certain novelty).

In conclusion, WHP significantly improves the level of aging-related lipid peroxidation and protein oxidation, improves the body’s multiple antioxidant enzyme activities, resists aging-related oxidative stress damage, and improves the potential of the body’s endogenous antioxidant defense mechanism. Moreover, WHP can significantly improve the decline of aging-related spatial exploration, body movement, and spatial and non-spatial learning and memory ability. Its specific mechanism may be related to reducing degeneration of hippocampal nerve cells, reducing apoptosis of nerve cells, improving cholinergic function, reducing the expression of inflammatory factors in brain tissue, reducing oxidative stress injury, and improving the expression of synaptic plasticity protein.

## 5. Conclusions

The above research results suggests that WHP can slow aging-related oxidative stress damage, and that it has a significant protective effect on learning/memory dysfunction induced by aging. WHP significantly improves the level of aging-related lipid peroxidation and protein oxidation, improves the body’s antioxidant enzyme activities, resists aging-related oxidative stress damage, and improves the potential of the body’s endogenous antioxidant defense mechanisms. The protective effect of WHP on brain aging may be related to reducing degeneration and apoptosis of hippocampal neurons, improving cholinergic function, reducing the expression of inflammatory factors in brain tissue, reducing oxidative stress injury, and improving the expression of p-CaMKⅡ and the BDNF synaptic plasticity protein. In addition, we found that the optimal dose of WHP supplementation in mice was 1.5 g/kg body weight, which showed a better protective effect on the D-galactose-induced aging model, but further experimental verification is needed. This study first demonstrated the role of WHP in alleviating aging-related oxidative stress injury and learning/memory impairment, providing an important “prospect” for the use of WHP as a sort of nutrient in brain aging. Further studies are needed to evaluate the clinical protective effect of WHP and determine the optimal dose of WHP supplementation in the human body.

## Figures and Tables

**Table 1:** Amino acid composition of WHP.

Amino Acid	Amino Acid Composition of WOPs (g/100 g)
Glu	14.4
Asp	8.9
Leu	8.6
Lys	8.1
Thr	5.8
Ile	5.4
Pro	5
Val	4.6
Ala	4.3
Ser	4.2
Phe	2.6
Tyr	2.5
Arg	2.3
Cys	2
Met	1.8
Trp	1.7
Gly	1.5
His	1.5
Hyp	<0.1

Glu, glutamic acid; Asp, Aspartic acid; Leu, leucine; Lys, lysine; Thr, threonine; Ile, isoleucine; Pro, proline; Val, valine; Ala, alanine; Ser, serine; Phe, phenylalanine; Tyr, tyrosine; Arg, argnine; Cys, cysteine; Met, methionine; Trp, tryptophane; Gly, glycine; His, hlstidine; Hyp, hydroxyproline.

**Table 2:** Effects of WHP on the space exploration capability of the open-field test.

Groups	No. of Squares Crossed	No. of Rearing	No. of Inner Squares Entered	Time Spent in Inner Squares (s)	No. Fecal Boli
Control	108.25 ± 18.59	18.13 ± 4.94	2.38 ± 1.51	1.31 ± 0.88	0.88 ± 1.13
Model control	45.38 ± 27.83 ^a^	5.50 ± 4.11 ^a^	0.88 ± 0.99	0.88 ± 0.83	1.13 ± 1.13
Whey protein	94.88 ± 34.71	13.25 ± 6.16 ^b^	2.25 ± 2.12	1.50 ± 1.20	0.63 ± 0.92
WHP-LG	74.13 ± 14.09	13.63 ± 8.68 ^b^	1.13 ± 1.81	15.88 ± 42.09	1.63 ± 1.60
WHP-MG	98.25 ± 26.18 ^b^	14.00 ± 10.41 ^b^	1.38 ± 1.19	1.00 ± 0.76	1.50 ± 1.41
WHP-HG	121.38 ± 47.88 ^b^	15.63 ± 8.67 ^b^	2.25 ± 0.89	1.63 ± 0.52	1.00 ± 1.20

Data are expressed as means ± SD (*n* = 8). ^a^ *p* < 0.05 indicates significant difference versus the control group; ^b^ *p* < 0.05 indicates significant difference versus the model group. Whey protein group at a dose of 1.5 g/kg; WHP-LG, whey protein peptide low dose group at a dose of 0.3 g/kg; WHP-MG, whey protein peptide medium dose group at a dose of 1.5 g/kg; WHP-HG, whey protein peptide high dose group at a dose of 3.0 g/kg.

**Table 3:** Effects of WHP on the learning and memory capability of the place navigation test.

Groups	Day 1			Day 3			Day 6		
	Escape Latency (s)	Distance Swam (cm)	Swimming Velocity (cm/s)	Escape Latency (s)	Distance Swam (cm)	Swimming Velocity (cm/s)	Escape Latency (s)	Distance Swam (cm)	Swimming Velocity (cm/s)
Control	40.64 ± 13.10	509.73 ± 164.36	12.88 ± 0.51	32.63 ± 17.25	523.95 ± 268.22	15.85 ± 0.93	26.30 ± 11.68 *	395.50 ± 161.89	15.28 ± 1.42
Model control	50.96 ± 4.99	570.67 ± 156.57	11.53 ± 0.90 ^a^	44.54 ± 10.22	599.31 ± 146.36	13.22 ± 0.86 ^a^	41.81 ± 12.16 ^a^	528.46 ± 140.85	13.07 ± 2.22 ^a^
Whey protein	45.43 ± 6.43	595.84 ± 80.27	12.88 ± 0.68 ^b^	35.88 ± 13.02	485.65 ± 160.29	13.50 ± 1.31	30.36 ± 13.88 *	369.74 ± 151.90	12.45 ± 1.14
WHP-LG	45.56 ± 10.50	558.95 ± 142.10	12.18 ± 0.87	36.09 ± 9.84	490.71 ± 127.12	13.72 ± 0.71	28.78 ± 10.13 *	372.64 ± 120.23	12.82 ± 0.95
WHP-MG	46.69 ± 12.20	553.57 ± 131.80	12.03 ± 0.83	38.02 ± 7.56	483.72 ± 99.15	12.86 ± 0.77	35.13 ± 17.79 *	414.54 ± 188.15	11.94 ± 1.20
WHP-HG	44.30 ± 10.38	532.32 ± 108.29	11.85 ± 0.77 ^c^	35.19 ± 16.06	423.73 ± 185.42	12.47 ± 0.92	31.38 ± 9.68 *	373.62 ± 85.40	12.11 ± 1.30

Data are expressed as means ± SD (*n* = 6). ^a^ *p* < 0.05 indicates significant difference versus the control group; ^b^ *p* < 0.05 indicates significant difference versus the model group; ^c^ *p* < 0.05 indicates significant difference versus the whey protein group; * *p* < 0.05 indicates significant difference versus the escape latency on day1.Whey protein group at a dose of 1.5 g/kg; WHP-LG, whey protein peptide low dose group at a dose of 0.3 g/kg; WHP-MG, whey protein peptide medium dose group at a dose of 1.5 g/kg; WHP-HG, whey protein peptide high dose group at a dose of 3.0 g/kg.

**Table 4:** Effects of WHP on the learning and memory capability of the spatial probe test.

Groups	Escape Latency (s)	No. of Platform Crossed	Time Spent in the Target Quadrant (s)	Distance in Target Quadrant (cm)
Control	17.61 ± 8.38	4.83 ± 1.72	12.15 ± 3.93	244.40 ± 68.63
Model control	45.48 ± 20.71 ^a^	1.00 ± 1.27 ^a^	6.16 ± 2.61a	108.96 ± 46.59 ^a^
Whey protein	40.54 ± 18.79	2.00 ± 2.45	10.15 ± 5.68	212.91 ± 131.77 ^b^
WHP-LG	40.15 ± 20.28	2.00 ± 1.90	9.30 ± 3.73	179.64 ± 71.95
WHP-MG	33.08 ± 22.37	1.50 ± 1.38	9.52 ± 3.37	199.11 ± 64.69
WHP-HG	22.86 ± 20.32 ^b^	2.67 ± 2.58	14.24 ± 5.69b	258.94 ± 111.32 ^b^

Data are expressed as means ± SD (*n* = 6). ^a^ *p* < 0.05 indicates significant difference versus the control group; ^b^ *p* < 0.05 indicates significant difference versus the model group. Whey protein group at a dose of 1.5 g/kg; WHP-LG, whey protein peptide low dose group at a dose of 0.3 g/kg; WHP-MG, whey protein peptide medium dose group at a dose of 1.5 g/kg; WHP-HG, whey protein peptide high dose group at a dose of 3.0 g/kg.

## Data Availability

The data presented in this study are available on request from the corresponding author. The data are not publicly available due to privacy.
